# Advances in the Diagnosis of Human Opisthorchiasis: Development of *Opisthorchis viverrini* Antigen Detection in Urine

**DOI:** 10.1371/journal.pntd.0004157

**Published:** 2015-10-20

**Authors:** Chanika Worasith, Christine Kamamia, Anna Yakovleva, Kunyarat Duenngai, Chompunoot Wangboon, Jiraporn Sithithaworn, Nattaya Watwiengkam, Nisana Namwat, Anchalee Techasen, Watcharin Loilome, Puangrat Yongvanit, Alex Loukas, Paiboon Sithithaworn, Jeffrey M. Bethony

**Affiliations:** 1 Department of Parasitology, Faculty of Medicine, Khon Kaen University, Khon Kaen, Thailand; 2 Liver Fluke and Cholangiocarcinoma Research Center, Faculty of Medicine, Khon Kaen University, Khon Kaen, Thailand; 3 Department of Microbiology, Immunology and Tropical Medicine, and Research Center for Neglected Diseases of Poverty, School of Medicine & Health Sciences, George Washington University, Washington, D.C., United States of America; 4 Department of Public Health, Faculty of Science and Technology, Phetchabun Rajabhat University, Phetchabun, Thailand; 5 Biomedical Science Program, Graduate School, Khon Kaen University, Khon Kaen, Thailand; 6 Faculty of Associated Medical Sciences, Khon Kaen University, Khon Kaen, Thailand; 7 Department of Pre-Clinical Veterinary Sciences, Faculty of Veterinary Sciences, Mahasarakham University, Mahasarakham, Thailand; 8 Department of Biochemistry, Faculty of Medicine, Khon Kaen University, Khon Kaen, Thailand; 9 Centre for Biodiversity and Molecular Development of Therapeutics, Queensland Tropical Health Alliance, James Cook University, Cairns, Queensland, Australia; Australian National University, AUSTRALIA

## Abstract

**Background:**

Many strategies to control opisthorchiasis have been employed in Thailand, but not in the other neighbouring countries. Specific control methods include mass drug administration (MDA) and health education to reduce raw fish consumption. These control efforts have greatly shifted the epidemiology of *Opisthorchis viverrini* (OV) infection over the last decade from presenting as densely concentrated "heavy" infections in single villages to widespread "light" OV infections distributed over wide geographical areas. Currently, the "gold standard" detection method for OV infection is formalin ethyl-acetate concentration technique (FECT), which has limited diagnostic sensitivity and diagnostic specificity for light OV infections, with OV eggs often confused with eggs of minute intestinal flukes (MIFs) in feces. In this study, we developed and evaluated the diagnostic performance of a monoclonal antibody-based enzyme-linked immunosorbent assay for the measurement of OV excretory-secretory (ES) antigens in urine (urine OV-ES assay) for the diagnosis of opisthorchiasis compared to the gold standard detection FECT method.

**Methodology:**

We tested several methods for pre-treating urine samples prior to testing the diagnostic performance of the urine OV-ES assay. Using trichloroacetic acid (TCA) pre-treated urine, we compared detection and quantification of OV infection using the urine OV-ES assay versus FECT in OV-endemic areas in Northeastern Thailand. Receiver operating characteristic (ROC) curves were used to determine the diagnostic sensitivity and specificity of the urine OV-ES assay using TCA pre-treated urine, and to establish diagnostic positivity thresholds. The Positive Predictive Value as well as the likelihood of obtaining a positive test result (LR+) or a negative test result (LR-) were calculated for the established diagnostic positivity threshold. Diagnostic risks (Odds Ratios) were estimated using logistic regression.

**Results:**

When urine samples were pre-treated with TCA prior to use in the urine OV-ES assay, the analytical sensitivity was significantly improved. Using TCA pre-treatment of urine, the urine OV-ES assay had a limit of detection (LoD) of 39 ng/ml compared to the LoD of 52 ng/mL reported for coprological antigen detection methods. Similarly, the urine OV-ES assay correlated significantly with intensity of OV infection as measured by FECT. The urine OV-ES assay was also able to detect 28 individuals as positive from the 63 (44.4%) individuals previously determined to be negative using FECT. The likelihood of a positive diagnosis of OV infection by urine OV-ES assay increased significantly with the intensity of OV infection as determined by FECT. With reference to FECT, the sensitivity and specificity of the urine OV-ES assay was 81% and 70%, respectively.

**Conclusion:**

The detection of OV-infection by the urine OV-ES assay showed much greater diagnostic sensitivity and diagnostic specificity than the current "gold standard" FECT method for the detection and quantification of OV infection. Due to its ease-of-use, and noninvasive sample collection (urine), the urine OV-ES assay offers the potential to revolutionize the diagnosis of liver fluke infection and provide an effective tool for control and elimination of these tumorigenic parasites.

## Author Summary

Improved diagnostic methods for the detection of *Opisthorchis viverrini* (OV) infection in humans is required for effective surveillance and control of this food borne parasite and the prevention of OV-induced bile duct cancer (cholangiocarcinoma or CCA). In this study, a novel urinary antigen detection method was established for quantitative diagnosis of opisthorchiasis by a monoclonal antibody-based enzyme-linked immunosorbent assay (urine OV-ES assay). Analysis of paired feces and urine samples from 235 subjects in Don Chang sub-district in Khon Kaen Province, Northeast Thailand revealed 81% sensitivity and 70% specificity of the urine OV-ES assay when compared to the current gold standard diagnostic method. Moreover, levels of antigen detected by the urine OV-ES assay significantly correlated with intensity of OV infection (P< 0001), with and the proportion of antigen positive diagnosis associated with increasing intensity of infection. Forty four percent of individuals determined to be egg negative subjects by the gold standard method formalin ethyl-acetate concentration technique were positive by the urine OV-ES assay. The ease and noninvasiveness of urine sample collection and the high diagnostic accuracy of the urine OV-ES assay provide an alternative means for the diagnosis of human opisthorchiasis and facilitate the prevention and control of opisthorchiasis in resource limited setting of Southeast Asia.

## Introduction

*Opisthorchis viverrini* (OV) infection is a major public health problem in the Mekong River Basin region of Southeast Asia, especially in Thailand, the Lao People’s Democratic Republic (Lao PDR), Cambodia, and Vietnam [1, 2]. The clinical sequelae of chronic opisthorchiasis are several advanced hepatobiliary pathologies [3], the most concerning being advanced periductal fibrosis and intrahepatic cholangiocarcinoma (CCA) [4, 5]. Based on its strong association with CCA, OV has been classified as a Group I biological carcinogen by the World Health Organization’s International Agency on Research in Cancer [6]. As chronic OV infection has such a fundamental role in the induction of bile duct fibrosis and CCA, a comprehensive strategy to control and eliminate these neglected tropical diseases (NTDs) has been undertaken in Thailand over the last several decades. After a decade of mass drug administration (MDA) and public health educational efforts to control consumption of the raw fish intermediate host in which OV metacercariae encyst, the epidemiology of OV infection has changed dramatically. Initially presenting as densely concentrated heavy infections, OV infections today appear "light" and are spread across extensive geographical regions, especially in Northeastern Thailand, where these control program have been most effective. Hence, a detection method which is analytically sensitive yet also rapid and easy to apply is required to monitor this “shifting landscape” of OV infection [7].

Currently, the “gold standard” diagnostic for OV infection is the formalin ethyl-acetate concentration technique (FECT), which quantifies OV eggs in feces. The FECT method has several important drawbacks, including limited analytical sensitivity (A-Sn): i.e., light intensity infections can go undetected, requiring extensive fecal sampling over the course of days, which can be logistically onerous (if not impossible) in the resource limited settings where OV transmission is currently occurring. In addition, FECT has been shown in several studies [8] to have a limited analytical specificity (A-Sp), with OV eggs often confused with the eggs from minute intestinal flukes infection (MIFs) and accurate distinction, requires the presence of an experienced microscopist [9, 10]. Finally, advanced hepatobiliary pathologies from chronic opisthorchiasis such as biliary tract obstruction from bile duct fibrosis or primary biliary sclerosis, can obstruct the flow of eggs into the lumen and hence into feces, making the detection of light OV infection by coprological method nearly impossible [11]. Together, these limitations decrease the utility of FECT where OV transmission occurs in the Mekong Basin Region [8].

To address these limitations, several immunological and molecular assays have been developed to detect the presence of OV in feces, with varying diagnostic accuracy [3, 10, 12]. The molecular methods tend to be highly specific, but they often lack analytical sensitivity because of the presence of numerous PCR inhibitors in the feces [13]. Antigen detection in feces using a capture ELISA has also been developed and is a promising approach, as it is highly sensitive for light infections [14–16]. Monoclonal antibody (mAb)-based systems have been shown to be sufficient to detect OV adult secretory products from light infections, i.e., when only a few adult worms are present and when eggs cannot be detected in the feces in an animal model [16, 17]. However, these mAb-based antigen detection methods remain coprological assays; i.e., they require numerous fecal samples for analysis. Immunodiagnostic methods utilizing human serum or plasma are also useful for detecting OV-infection and the concomitant risk of the hepatobiliary pathologies [18, 19]. However, serodiagnostic methods require a blood draw, blood processing, cold chain refrigeration of sera or plasma, and trained phlebotomists, and usually prove logistically onerous and even unfeasible given the limited infrastructure of many of the laboratories in these research poor setting. Less invasive and easier to handle sample matrices such as saliva or urine are the ideal specimens to be collected for detecting OV in resource poor OV endemic setting in Southeast Asia [12].

Recently, our group has shown that parasite-specific IgG can be readily detected in urine of individuals with chronic opisthorchiasis [20] which has implications for the study of opisthorchiasis-induced hepatobiliary and renal abnormalities as well as the detection of people at risk of developing OV-induced CCA. The utility of urine samples, particularly ease and non-invasiveness of the collecting technique, has not been examined to date. In the current study, we developed and optimized an ELISA protocol to quantify the level of crude excretory-secretory (ES) OV antigen extract in urine samples and then assessed its diagnostic accuracy for the detection of OV-infection in field setting, with urine from individuals from OV endemic areas at variable infection intensities. We then compared the relationship between the detection of OV infection by our novel urine OV-ES assay and OV infection determined by the current “gold standard method” of FECT. This study is the first report on the performance of a method for urinary antigen detection for the diagnosis of opisthorchiasis.

## Materials and Methods

### Study participants and sample collection

The study began in December 2013 and ended in December 2014. Table 1 shows the sample sets used in the current manuscript. Sample Set 1 used to develop and Sample Set 2 to field-test the performance of the mAb-ELISA for OV-ES with TCA treated urine (urine OV-ES assay) compared to the current “gold standard” coprological technique FECT. Sample Set 3 was used to determine cross reactivity of the mAb-ELISA for OV-ES with individuals mono-infected with helminths.

**Table 1:** Sample sets used in the optimization verification and studies of cross reactivity of the urine *O*. *viverrini*-Excretory Secretory (OV-ES) assay.

Sample	N	OV+	OV-	Sampling Method	Objective
1	50	40	10^†^	Targeted for OV+ or OV-	Optimization of assay parameters
2^‡^	235	125	110	Population-based	Verification of diagnostic parameters
3	189	97	92	Targeted for OV+ for helminth infections*	Cross-reactivity studies

OV, *O*. *viverrini*

*Other helminths included *Strongyloides stercoralis*, minute Intestinal flukes (MIFs), hookworms, *Taenia* spp., *Echinostoma* sp. and *Trichuris trichiura*.

^†^Ten participants were enrolled from *O*. *viverrini* non-endemic areas in Khon Kaen province, Thailand.

^‡^A detailed demographic description of Sample Set 2 can be found in Table 2 below.

#### Sample Set 1

Table 1 shows the sample used to optimize the analysis of urine treatment for the urine OV-ES assay. It consisted of 22 males and 28 females (n = 50), who donated an adequate volume of urine and feces which were used to compare and optimize several protocols for the treatment of urine prior to OV-ES antigen detection by the urine OV-ES assay. Forty of the individuals, from an OV endemic area in Khon Kaen province had FECT confirmed OV infection and were designated as "positive controls" in this optimization phase. The remaining ten individuals, who had never resided in an OV endemic area (as determined by interview) and who were not infected with OV as confirmed by FECT were designated as the "negative controls" for this optimization phase. All OV infected individuals were treated for their infection with praziquantel.

#### Sample Set 2

The participants in sample set 2 were recruited during the baseline cross-sectional survey of a prospective study using a household sampling method from the Don Chang sub-district of Khon Kaen province, Northeast Thailand, which is an OV endemic area with a high incidence of OV-induced CCA. The epidemiology of OV transmission and CCA in this region has been as described in detail elsewhere [21, 22]. After providing informed consent, the participants were asked to supply single specimens of urine (5 mL) and fecal samples (10 grams) as well as demographic information to an interviewer. Of the 281 individuals recruited, only 235 individuals provided enough urine and fecal material to be enrolled in the study. There was no statistical difference in demographics (age, sex, etc.) from those who provided these samples and those who did not (S1 Fig). Tables 1 and 2 describe individuals who provided both fecal and urine samples to verify the applicability and diagnostic accuracy of the urine OV-ES assay in population based field setting.

**Table 2:** Characteristics of Sample Set 2 from endemic area of Don Chang, Khon Kaen Province.

Variable	Group				Total (%)
	1	2	3	4	
	OV Mono-infection (%)	OV Helminth co-infection (%)	Other helminth Infection (%)	Parasite negative (%)	
N	97	28	47	63	235
Sex					
Male	52 (54)	9 (32)	24 (51)	39 (62)	124 (53)
Female	45 (46)	19 (68)	23 (49)	24 (38)	111 (47)
Age					
Mean ± SD	55.82 ± 10.79	57.50 ± 9.42	50.14 ± 9.61	51.65 ± 10.57	53.1 ± 10.56
Interval					
≤ 30	0 (0)	0 (0)	0 (0)	1 (2)	1 (1)
31–40	4 (4)	0 (0)	6 (12)	2 (3)	12 (5)
41–50	30 (31)	6 (21)	21 (45)	24 (38)	81 (34)
>50	63 (65)	22 (79)	20 (43)	36 (57)	141 (60)
*O*. *viverrini* EPG*					
0	0 (0)	0 (0)	47 (100)	63 (100)	110 (47)
1–50	68 (71)	13 (46)	0 (0)	0 (0)	81 (34)
51–100	14 (14)	5 (18)	0 (0)	0 (0)	19 (8)
>100	15 (15)	10 (36)	0 (0)	0 (0)	25 (11)

*EPG, eggs per gram of feces

#### Sample Set 3

Table 1 shows the sample set used to determine the cross-reactivity of the urine OV-ES assay when applied to individuals with other endemic helminth infections. The samples were drawn from a specimen repository at the Department of Parasitology, Faculty of Medicine, Khon Kaen University (n = 92). Parasitic infections in these samples were confirmed by standard coprological examination methods as described below.

#### Fecal examination

The presence and the intensity of OV infection were determined by FECT with slight modification from the previous protocol [4, 5] as described below. Briefly, two grams of fresh feces were transferred from the sample into a tube containing 7 mL of 10% formalin. The samples were thoroughly homogenized and strained through two layers of gauze into the 15 mL centrifuge tube. The strained suspension was centrifuged at 2,500 rpm for 5 min and the supernatant was poured out. Ten mL of 0.85% saline were added to the tube and then mixed, followed by the addition of 3 mL of ethyl acetate to aid in the extraction of fat from the stool. The suspension was then centrifuged at 2,500 rpm for 5 min and resulted sediment was fixed with 1 mL of 10% formalin. The final fecal suspension was examined two time per sample by the same microscopist using a compound microscope at 40X with the results combined to calculate the number of eggs per gram of feces (EPG).

To confirm the presence of strongyloides and hookworm infection, the Agar Plate Culture Technique (APCT) as previously described by Koga [23] were performed. Briefly, 4 g of fecal sample was placed on a 1.5% nutrient agar plate and incubated at room temperature for 2–4 d. The presence of worms was assessed by washing the surface of each plate with 10 mL of 10% formalin, transferring the wash into a test tube, centrifuging, and examining the sediment under a microscope.

### Development of urine *O*. *viverrini* Excretory Secretory (OV-ES) assay

#### Urine collection, storage, and pre-treatment

First morning mid-stream urine samples were collected in plastic containers and kept in a 4–8°C box during transport to the laboratory, which occurred within 24 hours to ensure timely processing. Urine samples were centrifuged at 1,500 rpm at 4°C for 15 min and the clarified supernatants were aliquoted into screw top vials and stored at -20°C until used in the urine OV-ES assay with urine pre-treated by TCA, freezing, heating, or alkaline as discussed below.

#### Optimization of urine pre-treatment methods for urine *O*. *viverrini* Excretory Secretory (OV-ES) assay

Urine specimen from Sample Set 1 were further processed to remove interfering components by one of four treatment methods: (i) no treatment (i.e. the samples were frozen before being analyzed and served as untreated controls), (ii) heat treatment for 30 min at 70°C, (iii) alkaline treatment with an equal volume of 0.244 M carbonate buffer (pH 9.6) added to each urine sample and thoroughly mixed, and (iv) trichloroacetic acid (TCA) treatment with an equal volume of 4% TCA solution added to the sample and incubated for 20 min at room temperature and neutralized with an equal volume of 0.244 M carbonate buffer (pH 9.6) [24].

#### Coating antibodies for urine *O*. *viverrini* Excretory Secretory (OV-ES) assay

The monoclonal antibody (IgG1 murine mAb, clone KKU505), which has previously been shown to be reactive to excretory-secretory (ES) antigen extract from adult OV flukes (S1 Text) was used to coat the microtiter plates as part of the indirect sandwich ELISA. Both ES and somatic crude OV antigen extracts were prepared from short term *in vitro* culture of adult OV worms obtained from hamsters experimentally infected with OV metacercariae as previously described [25]. Flat-bottom 96-well microtiter plates (NUNC, Roskilde, Denmark) were coated with 5 μg/mL of the monoclonal antibody diluted in 50 mM bicarbonate buffer pH 9.6. The plates were sealed and then incubated overnight at 4°C and on the following day washed 3 times with 0.05% Tween20 in PBS pH 7.4 (PBST).

#### Treated urine applied to assay for OV-ES antigen capture

The plates were blocked with 200 μL of 5% skimmed milk powder in PBST and incubated at 37°C for 1 hour and washed again 3 times with PBST, followed by the addition of urine samples treated by one of the methods described in detail below at 100 μL/well in duplicates and incubated at 37°C for 2 hours.

#### Addition of 2^nd^Ab and biotinylated anti-species conjugate for urine *O*. *viverrini* Excretory Secretory (OV-ES) assay

Plates were washed 5 times with PBST, and 100 μL of a protein A purified rabbit IgG (10 μg/mL) against crude OV adult ES antigen extract was added to each well and incubated at 37°C for 2 hours. After washing three times, 1:4,000 diluted biotinylated-goat anti rabbit IgG (Invitrogen, CA, USA) in PBST was added and incubated at 37°C for 1 hour. Thereafter, the plates were washed 3 times with PBST, and 100 μL/well of streptavidin horseradish peroxidase (HRP) conjugate (Zymed, CA, USA) was added diluted 1:10,000 in PBST. After incubation for 30 min, the plates were washed 3X with PBST, and the substrate solution Orthophenylenediamine hydrochloride (Sigma, St. Louis, MO, USA) was added to wells and incubated for 20 min in the dark at room temperature. The reaction was stopped by the addition of 2M sulfuric acid (H_2_SO_4_), and the plates were read on an absorbance reader (Tecan, Austria) at the optical density (OD) at 492 nm.

Two well-trained laboratory personnel were responsible for execution of the sample analysis of both tested samples (index cases) and reference standard tests and they were analyzed simultaneously. During the sample execution, the sample IDs were blinded and the laboratory staffs had no knowledge of the sample subjects.

### Assessment of assay characteristics: Standard curve calibration and the Limit of Detection (LoD)

Negative urine samples from Sample Set 1 (n = 10) were “spiked” with varying concentration of crude OV adult ES antigen extract starting with 5000 ng concentration of OV-ES, two-fold serially diluted to produce a standard calibration curve (S2 Fig). A best-fit linear regression line was obtained from the serially diluted spiked urine. The regression line was utilized in each assay and functioned as the assay standard calibration curve from which antigen concentrations in urine samples were predicted by interpolation. To elucidate the limit of detection (LoD) of the assay, the same set of negative TCA treated urine samples were spiked with serially decreasing concentrations of OV-ES antigen. The highest concentration of spiked urine that interpolated below the value resulting from interpolating unspiked negative urine onto the standard calibration curve was considered the LoD of the assay.

### Identification of optimal urine treatment method and characterization of assay diagnostic parameters

Urine from Sample Set 1 was used to evaluate the performance of urine treatment methods in the assay as follows: initially, the OD values obtained from the urine OV-ES assay were transformed to a ratio between the OD of the samples and the OD of reference urine samples. Wilcoxon rank-sum test was performed to compare the distributions of OD values obtained from various treatment methods (freezing only (i.e., no treatment), heating, alkaline treatment, or TCA treatment); Bonferroni correction was used to adjust for multiple comparisons (Table 3). A Receiver Operating Characteristic (ROC) curve [26] comparing different urine treatment methods in the urine OV-ES assay was constructed by plotting the sensitivity against 1-specificity for the OD values detected using each urine treatment method on FECT confirmed positives and negatives. The Area under the Curve (AUC) was a measure used to determine the probability of correctly identifying an OV positive individual (as determined by FECT) as a True Positive and an OV negative individual (as determine by FECT) as a True Negative. The urine treatment methods that generated the ROC curve with the highest AUC was considered the optimal urine treatment method for use in the urine OV-ES assay.

**Table 3:** Sensitivity and specificity of ELISA treatment methods in the detection of *O*. *viverrini* positive individuals and difference by mean Optical Density (OD) value comparing urine pre-treatment methods.

**Treatment Methods**	**Sensitivity (%)**	**Specificity (%)**
* **TCA**	97.5	100
**Heating**	72.5	50.0
**Alkaline**	80.0	70.0
**Frozen**	95.0	70.0
**Difference in means comparing treatment methods to TCA**		
**Treatment methods**	**OD (492 nm) value (Mean ± SD)**	**p-value**
**TCA vs Frozen**	0.757 ± 0.19 vs 0.681 ± 0.17	<0.001
**TCA vs Alkaline**	0.757 ± 0.19 vs 0.516 ± 0.15	<0.001
**TCA vs Heating**	0.757 ± 0.19 vs 0.490 ± 0.14	<0.001

*TCA, trichloroacetic acid

### Determination of applicability and functional performance of the urine *O*. *viverrini* Excretory Secretory (OV-ES) assay in a field setting using Sample Set 2

Diagnostic sensitivity and diagnostic specificity, established from ROC curve analysis, were utilized to evaluate the performance of the urine OV-ES assay in an OV endemic area (Sample Set 2). To further characterize assay performance, the Positive Predictive Value (PPV) was calculated using a OV prevalence rate obtained from the field study which is comparable to the previous data from the OV endemic areas in the region [20, 27]. These values were also used to compare the diagnostic accuracy of the OV-ES assay, using TCA treated urine to the gold standard FECT. The threshold for diagnostic positivity was obtained by maximizing the sensitivity and specificity of each data point from the ROC curve. The relationship between OV infection status in this sample and urinary antigen concentrations, determined by the urine OV-ES assay, was investigated with a logistic regression model. The model was used to describe the association between elevated antigen levels and positive OV infection status; odds ratios (ORs), with corresponding 95% confidence intervals (CIs), are reported in the results and describe this relationship. A 0.05 significance level (alpha = 0.05) was utilized to determine meaningful predictors in the model.

### Cross reactivity studies

In an attempt to identify potential sources of false positivity, urine from OV-negative individuals with other helminths infections (Sample Set 3) was treated with TCA and infection status was evaluated with the OV-ES urine antigen assay. The other helminths infections in this study sample included *Strongyloides stercoralis* (n = 56), minute intestinal flukes (MIFs) (n = 13); hookworms (n = 10); *Taenia* sp. (n = 6); *Echinostoma* sp. (n = 4); and *Trichuris trichiura* (n = 3). A false positive diagnosis resulted when a values from the urine of an individual infected with one of these helminths was above the cutoff value established from sample set 2.

### Ethical statement

This human subject protocol was approved by the Human Ethics Committee of Khon Kaen University, Thailand (reference number HE561478) and written informed consent was obtained from individual subjects and those with parasite-positive examination by FECT were treated with anthelmintic drugs.

The experimental protocol for monoclonal antibody production was approved by the Institutional Animal Ethical Committee, Khon Kaen University (AEKKU-NELAC 26/2557) and was performed in strict accordance with the guideline for the Care and Use of Laboratory Animals of the National Research Council of Thailand.

### Data analysis and statistical methods

Statistical analyses were performed using SPSS 21 (IBM, Chicago, IL, USA) and SAS 9.3 (Cary Institute, NC, USA). Kendall’s tau-b correlation test was used to determine the correlation between urinary antigen concentration and EPG. The usefulness of urinary antigen, detected by mAb-ELISA for diagnosis of opisthorchiasis, was evaluated in a multiple logistic regression model. Diagnostic accuracy of the mAb-ELISA for OV ES using TCA treated urine in terms of clinical sensitivity, clinical specificity, and the positive predictive values and negative predictive values were estimated by Receiver Operation Curve (ROC) curve analysis using MedCal (MedCalc Software, Ostend, Belgium). Statistically significant level was set as p<0.05.

## Results

### Performance of urine pre-treatment methods for urine *O*. *viverrini* Excretory Secretory (OV-ES) assay using Sample Set 1

**Fig 1:**
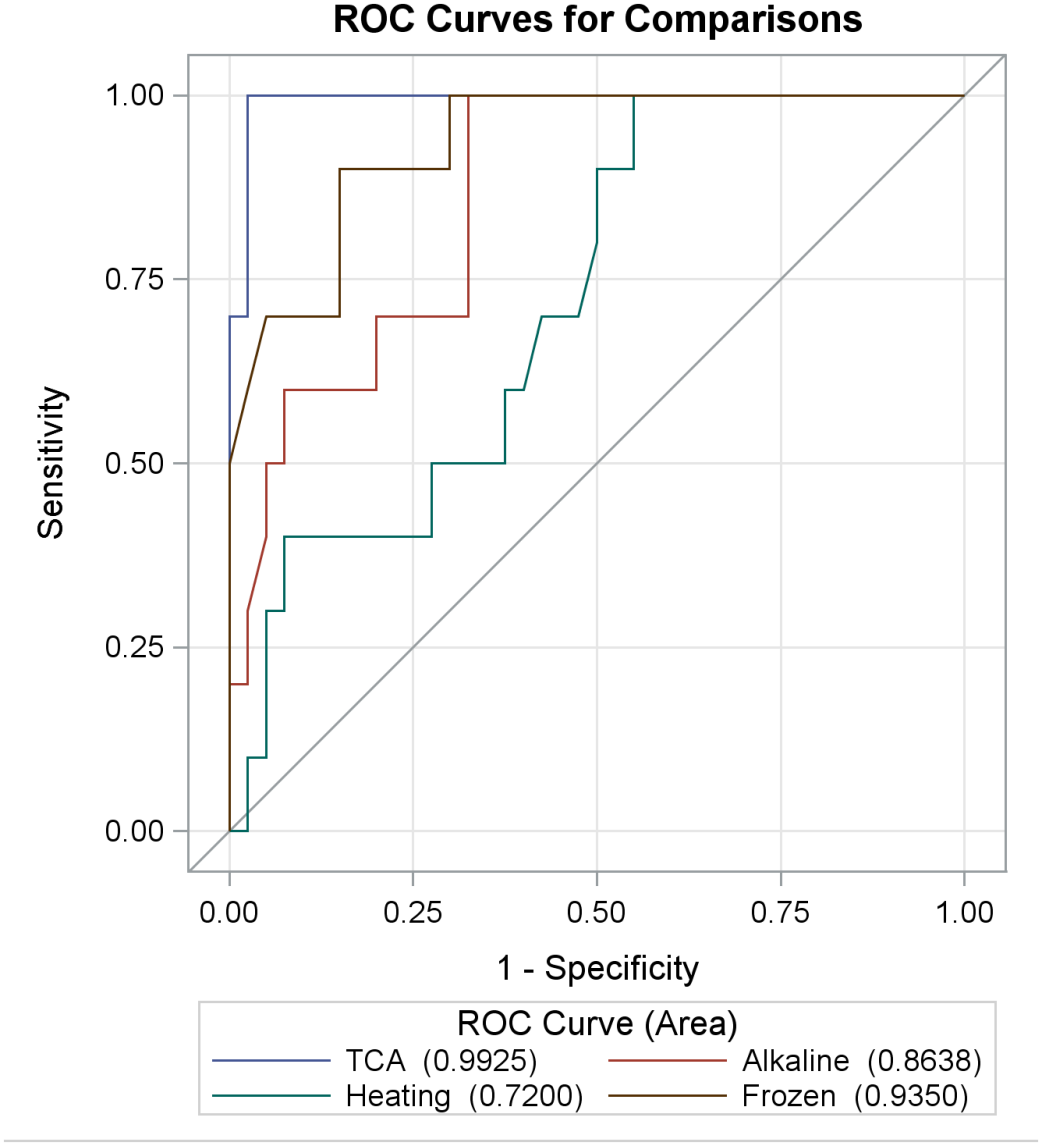
Receiver operating characteristic (ROC) curve analyses comparing trichloroacetic acid (TCA), frozen, heating and alkaline methods in detecting *O*. *viverinni* infection in Sample Set 1 (n = 50). ROC curve, using individuals from Sample Set 1 were used to compare the diagnostic performance of antigen detection TCA, frozen, heating and alkaline treatment methods in the *O*. *viverrini* Excretory Secretory assay. Sensitivity against 1-specificity for the antigen levels detected by each ELISA method from confirmed positives and negatives by the gold standard method formalin ethyl-acetate concentration technique (FECT).

As shown in Fig 1, the TCA method produced an ROC curve with the highest AUC of 0.9925. The sensitivity and specificity for each urine treatment method are reported in Table 3. Samples treated by the TCA method increased the OD range of the assay as shown in Table 3, i.e., treating urine with TCA produced the highest OD levels in the urine OV-ES assay compared to the other treatment methods.

### Receiver operating characteristic (ROC) curve analyses showed that trichloroacetic acid (TCA) was better than pretreatment method for urine to detect OV infection in the *O*. *viverrini* Excretory Secretory (OV-ES) assay

Fig 1 and Table 3 show the ROC curve analysis using individuals from Sample Set 1 (n = 50) to compare the diagnostic performance of antigen detection when urine is pretreated using TCA, freezing (-20°C), heating at 70°C, or alkaline before use in urine OV-ES ELISA. The sensitivity against 1-specificity for the antigen levels detected by each ELISA method from confirmed positives and negatives by the gold standard method (FECT).

#### Analytical sensitivity of the assays

As expected, the OD values at 492 nm in spiked urine samples were higher than the corresponding negative controls (S2 Fig). The minimum urine OV-ES antigen concentration measured by the TCA treated urine when used in the urine OV-ES assay was 39 ng/mL. There were positive linear relationships between concentrations of spiked ES antigen of *O*. *viverrini* and OD values obtained by the urine OV-ES assay. The best-fit linear regression equation was y = 0.941x-0.914 (y = OD, x = antigen concentration) and this equation subsequently was used to calculate the concentration of antigen in analyses of field collected urine samples.

### Field application of the urine *O*. *viverrini* Excretory Secretory (OV-ES) assay using trichloroacetic acid (TCA) treated urine

**Fig 2:**
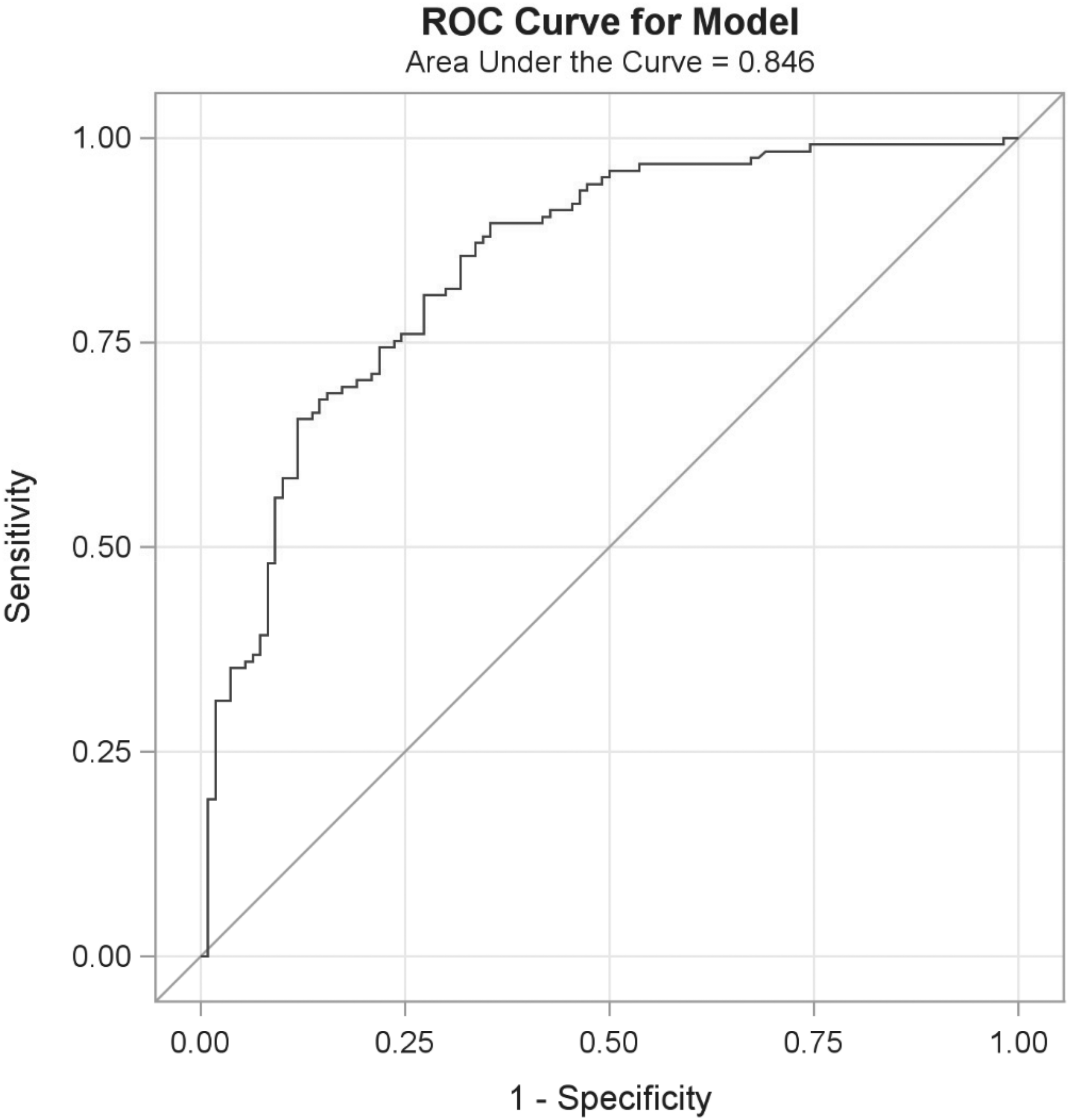
Receiver operating characteristic (ROC) curves comparing urine *O*. *viverrini* excretory secretory (OV-ES) assay method to the gold standard formalin ethyl-acetate concentration technique (FECT) (n = 235). The ROC curve illustrates the diagnostic performance of antigen detection using the urine OV-ES assay compared to the gold standard FECT method. The assay had AUC of 0.8460. The ROC curve diagnostic sensitivity was modeled as included negative controls (OV negative and other infections) and diagnostic sensitivity were modeled as individuals who were OV+ with and without co-infections.

Table 4 shows the threshold to obtain the diagnostic cutoff for positivity (i.e., OV infection) using the urine OV-ES assay as determined by the point on the ROC curve (Fig 2) where the diagnostic sensitivity (D-SN) and diagnostic specificity (D-SP) were concurrently maximized. The positive and negative predictive values (PPV, NPV) and positive and negative likelihood ratios (LR+, LR-) were estimated based upon a prevalence of OV of 50% are also presented in Table 4. It should be noted that despite the fact that the D-SN and the D-SP decreases when applied in the field setting decreases, the assay’s diagnostic performance remains robust with an AUC of 0.846,a PPV of 75%, NPV of 76%, and a LR+ of 2.69 and LR- of 0.27. Moreover, despite being developed in a small and targeted sample set (Sample Set 1), the application to a larger, randomly selected group (Sample Set 2) verified the diagnostic performance of the urine OV-ES assay (S1 Table). A logistic regression model was applied to this data set to determine the odds of having a positive diagnosis based on increasing OV-ES urine antigen levels as measured by the urine OV-ES assay and are presented in Table 4. It should be noted that a one unit increase in urine OV as detected by the urine OV-ES assay has an increased odds of having OV of 9% while 10 unit increase urine OV as detected by the urine OV-ES assay has an increased odds of having OV of 230%.

**Table 4:** Diagnostic performance of antigen detection by the urine *O*. *viverrini* Excretory Secretory (OV-ES) assay compared with the gold standard formalin ethyl-acetate concentration technique (FECT) in field-collected samples (n = 235) and the odds on predicting opisthorchiasis by urinary antigen detection using urine OV-ES assay.

**Comparator**	**Diagnostic performance of urine OV-ES assay**							
	AUC*	Cutoff	Sensitivity	Specificity	°PPV	°NPV	° ^†^LR+	° ^†^LR-
FECT	0.846	36.5	0.81	0.70	0.75	0.76	2.69	0.27
**Predicting OV-infection by urine OV-ES assay**								
**Unit increase urine antigen**			**Odds Ratio**		**95% Confidence Interval**			
					**Lower**		**Upper**	
1			1.088		1.056		1.120	
10			2.319		1.771		3.176	
20			5.357		3.137		10.087	
30			12.464		5.557		32.036	

*AUC refers to the area under the Receiver operating characteristic (ROC) curve.

°Positive Predictive Value (PPV), Negative Predictive Value (NPV) and Likelihood Ratios (LR) were estimated using 53% prevalence rate of OV.

^**†**^The term LR+ refers to the likelihood of observing a positive test result in patients with OV;

LR- refers to the likelihood, after subtracting from 1, of observing a negative test result with individuals without OV infection

### Cross reactivity testing for the urine *O*. *viverrini* Excretory Secretory (OV-ES) assay

**Fig 3:**
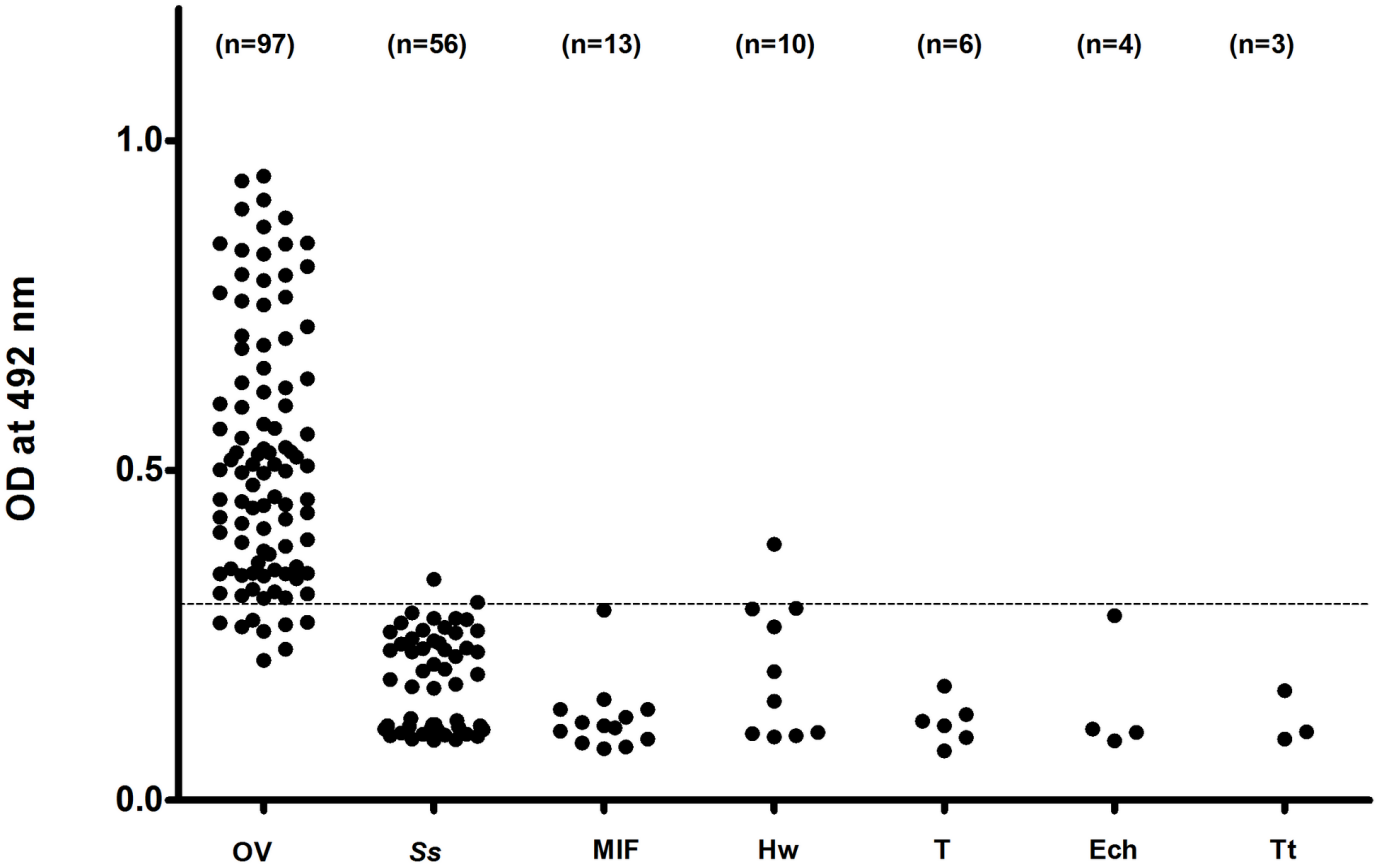
Cross reactivity with other helminthes of using the urine *O*. *viverrini* Excretory Secretory (OV-ES) assay with trichloroacetic acid (TCA) pre-treated urine. Most helminth infections resulted in negative tests except for 2 of 56 *S*. *stercoralis* infections (3.57%) and 1 of 10 hookworm infections (10%). Data points shown are antigen levels (OD 492) of individuals infected with OV, *O*. *viverrini*; Ss, *S*. *stercoralis*; MIF, minute intestinal flukes; Hw, hookworms; T, *Taenia*; Ech, Echinostomes and Tt, *Trichuris trichiura*.

Based on urine samples from participants in Sample Set 2 (Table 1), tests for cross reactivity of the urine OV-ES assay for other helminth infections endemic to the area were used to evaluate the analytical specificity of the assay as shown in Table 3. Most yielded negative tests except for 2 of 56 (3.57%) subjects with *S*. *stercoralis* infections, and 1 of 10 subjects with hookworm infections (10%) as seen in Fig 3.

## Discussion

The gold standard method for detecting OV infection is the coprological technique FECT, which is relatively sensitive for medium to heavy OV infection, but lacks analytical sensitivity for light OV infections. As shown in recent several studies, even light OV infection remains strongly associated with significant hepatobiliary pathology, such as advanced periductal fibrosis and CCA, for which this food borne trematode is best known [28]. Moreover, the FECT also lacks analytical and diagnostic specificity (especially against the MIFs which are endemic in Mekong Basin Region), and can be logistically challenging, especially in the resource-poor settings of Southeast Asia, where OV is the most prevalent and its concomitant morbidity and mortality from intrahepatic bile duct cancer or CCA is highest in the world [29]. The coprological techniques for OV are especially onerous given the changing distribution of OV infection due to mass drug administration (MDA), with the distribution of OV infection going from densely clustered medium to heavy infections to widely dispersed light OV infections, but with the rates of hepatobiliary pathology, especially CCA, remaining the highest in the world. Mass Drug Administration using praziquantel for opisthorchiasis in Thailand commenced in 1984, when the prevalence of OV was on average 63%, with MDA continuing until 2001 which brought the average prevalence of OV down to 9.3% [1]. As such, the current coprological methods for the detection of OV infection are insufficient for this new “landscape” of OV-infection. Herein, we propose a urine-based detection method by the urine OV-ES assay, which utilizes advances already made in the serological testing for OV, but applied to pretreated urine samples. Currently, the serological diagnosis of OV is problematic due to the invasiveness of blood draw, cold chain requirements, a lack of analytical and diagnostic specificity, and the persistence of antibodies even after treatment for OV infection, making the determination of an active OV infection from a former OV infection impossible. However, the monoclonal antibodies used in the serological assays can be modified for use in alternative approaches to diagnose active OV infection: i.e., urine OV-ES assay. Here, we adopted antibody approach using the non-invasive sample matrix (urine) in an easily applied assay that could be used to determine OV infection in these new epidemiological circumstances.

In our study, the effects of four treatment protocols on urine specimens were compared. The TCA-treated urine yielded higher OD values in the urine OV-ES assay, with a greater discriminatory power between “known OV positive” and “known OV negative” samples than when urine was treated by freezing, heating, or alkaline prior to use in the urine OV-ES assay. These data confirm findings in a recent copro-antigen analysis that also utilized anti-OV-ES antibodies for the detection of opisthorchiasis [17]. The pre-treatment with TCA has been also successfully added to serum and urine samples to precipitate out the interfering proteins for successful antigen detection in schistosomiasis [24, 30, 31].

In the present study, LoD of the urine OV-ES assay was 39 ng/mL, which is better than the copro-antigen detection using this same monoclonal antibody of 52 ng/mL as previously reported [17]. The greater analytical sensitivity of the urine OV-ES assay is not surprising since the same monoclonal antibody clone (clone KKU505) and only a slightly different urine OV-ES assay protocol were used in the current study from that of Watwiengkam and colleagues [17]. In regards to the greater specificity of the urine OV-ES assay, cross reactivity with urine from OV-negative individuals with other parasitic infections endemic to the region was observed in only 2 of the 56 subjects infected with *S*. *stercoralis* (3.57%) and in only 1 from 10 subjects infected with hookworm (10%). It is likely that these cases may represent mixed infections of *S*. *stercoralis* or hookworm with low intensity *O*. *viverrini* infection, or that *O*. *viverrini* was a recent infection in the pre-patent period where eggs are yet to be detected. Furthermore, the results confirmed that there was no cross reactivity with other helminth parasites endemic to the area since the levels of antigen in subjects with *O*. *viverrini* infection alone or with mixed infection with other parasites (i.e. *S*. *stercoralis*, echinostomes, hookworm, minute intestinal fluke and *Taenia*) were not different. However, further tests of urine samples from non-endemic areas of opisthorchiasis are required to ensure the lack of this observed cross reactivity.

In this study, analyses of quantitative urinary-antigen levels revealed that the intensity of OV infection was significantly correlated with the concentration OV-ES antigen in urine. This finding was similar to copro-antigen levels and intensity of infections with both OV [17] and experimental clonorchiasis [32]. Furthermore, a significant correlation has also been found between egg counts and levels of *S*. *haematobium* antigen in urine samples [33]. Therefore, the measurements of antigen level in feces and urine samples provide an advantage not only for diagnosis of light infection but also for the diagnosis of the intensity of OV infection. Whether the urinary antigen associates with severity of disease or not as observed in other diseases [34–38] is the subject of future investigations by our group.

In comparison with copro-antigen detection in opisthorchiasis reported previously by Watwiengkam et al [17], urinary antigen level was approximately 25 times lower than copro-antigen at infection intensities of 1–100 EPG. The explanation for this discrepancy is probably due to the fact that OV adult worms secrete antigen directly into the bile duct before being passively swept into the gastrointestinal tract and voided in the feces as copro-antigens. By contrast, using our urinary antigen detection method, the parasite antigen may be sequestrated or trapped in the liver or in other tissue, and diffused into the circulation before being excreted via the kidneys with urine. Therefore, unknown quantities of OV-ES antigen may be trapped in tissue with residual amounts are excreted into urine. Whether levels of urinary antigen detected have any relationship to kidney pathology as observed previously by our group with antibody to *O*. *viverrini* in urine is not known [20]. It worthy to note that, although very little underlying information is available, the fact that we are able to detect OV-ES antigen in urine specimens forms the basis for further studies to elucidate the mechanisms involved in the antigen secretion pathway of infected individuals, as well as its contribution in hepatobiliary pathology and formation of CCA.

With reference to the gold standard diagnosis of FECT, performance of the urine OV-ES assay on field samples yielded a high diagnostic sensitivity and diagnostic specificity of 81% and 70%, respectively. The prevalence of OV by antigen detection (64%) was higher than that determined by FECT (53%). The main difference was due to the finding of a considerable number of antigen positive cases (28 out of 63 cases, 44%) that were deemed the egg-negative using FECT. Autopsy data have shown that a considerable number of egg-negative cases (70%) can occur in individuals with worm loads <20 [39], which suggests that the current gold standard FECT diagnosis may not be ideal and caution is needed when interpreting the data generated by this method. Another possibility is that the result of examinations using only a single fecal sample may underestimate the presence of light infection in endemic populations multiple fecal samples may help to increase the sensitivity of fecal examination [40]. Nevertheless, without a reliable gold standard method, a repeat of a specific method or combination of methods is required; for example, a combination of fecal examination and serological tests has been suggested for more accurate diagnosis of clonorchiasis [41]. Moreover, it is not feasible to detect eggs in cases of opisthorchiasis in the presence of biliary obstruction due to periductal fibrosis or in the case of low infection intensity [42]. Each infected person in this study was likely at a different stage of infection, particularly when worms are still immature and therefore eggs were not detectable by FECT. In this situation, metabolic products may be secreted in considerable quantity from the parasites such that it is possible to be readily detected by the urine OV-ES assay.

Interestingly, we found that among the 97 cases from egg-positive individuals, antigens were detected only in 89 cases while 8 cases were found to be negative following urine OV-ES assay. These subjects had low EPG (EPG 7–19), which may be a problem with false-negative results. In addition to worm burden, the concentration of OV antigens is influenced by many other factors, such as duration and degree of biliary fibrosis, or the formation of circulating immune complexes that are difficult to detect [43].

In order to improve the diagnostic accuracy even more when the OV-ES assay is used in low transmission scenarios, one possibility is to modify the current protocol to increase volume of the urine sample in the assay. In the current protocol, we used 100 μL of sample diluted in 4% TCA, with only 50 μL of urine sample was used. A similar strategy has been successfully investigated in detecting circulating antigen in urine in Asian schistosomiasis (*Schistosoma japonicum*) in which the results showed that by increasing volume of urine sample from 250 μL to 2 mL of urine, the diagnostic sensitivity has increased 5 folds [44, 45].

It is known that the gold standard diagnostic method by conventional fecal examination i.e. FECT has limitation in diagnostic accuracy [8]. However, the OV-ES assay in the current format also has a potential drawback in that it requires sophisticated instrumentation (i.e., an microplate reader), reagents, and well-trained technical staff. However, these drawbacks can be mitigated if the OV-ES assay is further developed into a simplified strip test kit for point of care (POC) use similar to that for schistosomiasis [46]. More studies on larger sample population, more tests in communities with different endemic settings and more tests with concurrent trematode infections i.e. schistosomiasis are needed.

In conclusion, our results show that the urine OV-ES assay can be an effective tool for urinary-antigen detection in opisthorchiasis by the incorporation of a TCA urine treatment protocol, and strongly suggest that TCA-treated urine is a good alternative for detection of ES antigens of *O*. *viverrini*. Urine antigen and intensity of *O*. *viverrini* infection levels were strongly correlated. The advantage of using urine samples is the non-invasive ease of collection as well as high acceptability by individuals and the community. Moreover, this protocol has offered high sensitivity and specificity, which is essential for the effective surveillance and control of opisthorchiasis. Further studies are required to evaluate the performance and utility of urinary antigen detection for diagnosis of *O*. *viverrini* and to assess the effect of chemotherapy in opisthorchiasis.

## Supporting Information

S1 Text Production of monoclonal antibody. (DOCX)

S1 Table Diagnosis of *O*. *viverrini* by FECT and OV-ES assay of TCA-treatment samples in different sample sets. (DOCX)

S1 Fig Flow diagram of participants and parasitic infection rates determined by formalin ethyl-acetate concentration technique (FECT) and urine OV-ES assay. N refers to the number of participants. The abbreviation “OV” refers to *O*. *viverrini*. Other parasites include *S*. *stercoralis*, Minute intestinal fluke (MIF), Hookworm, *Echinostoma* spp. **and** *Taenia* sp.
(PDF)

S2 Fig Relationship between OD values and *O*. *viverrini* antigen in urine. Data shown are optical density (OD) at 492 nm from OV-ES assay of TCA treated urine specimens against varying concentration of OV-ES antigen compared with negative control.
(TIF)

S1 Checklist STARD Checklist. (DOCX)
