# Supplementary material for: Advances in the Diagnosis of Human Opisthorchiasis: Development of Opisthorchis viverrini Antigen Detection in Urine
Source: PLoS Negl Trop Dis. 2015 Oct 20;9(10):e0004157. doi: 10.1371/journal.pntd.0004157 (PMC4618926; doi:10.1371/journal.pntd.0004157)
Supplement: S1 Text — (DOCX) [file pntd.0004157.s001.docx]

**S1 Text. Production of monoclonal antibody.**

The production of monoclonal antibody to *Opisthorchis viverrini* excretory-secretory antigen (OV-ES antigen) was performed following methods previously described by Amornpunt et al [1] and Billing et al [2]. In brief, the crude excretory-secretory (ES) antigen extracted from *O. viverrini* was prepared from short term *in vitro* culture fluid of adult *O. viverrini* flukes. Spleen cells from BALB/c mice previously immunized with the OV-ES antigen were used for fusion with mouse myeloma cell line (clone P3x63-Ag8.653) to produce a hybridoma cell line. The resulting hybridoma were subjected to cloning by limited dilution method and the clones that secreted antibody to OV-ES antigen were picked and cloned again for the second time. The monoclonal antibody was checked for specificity to *O. viverrini* by checking its cross reactivity with a series of parasite crude parasite antigen extracts: *Fasciola gigantica, Taenia saginata, Spirometra sp., Dipylidium caninum, Angistrongylus cantonensis,* Hookworm, *Strongyloides stercoralis.* None of the clones had cross reactivity with crude antigen extracts from these parasites, all of which are endemic to Northeastern Thailand. The antigen recognition profile of OV-ES monoclonal antibody was determined by immunoblotting of OV-ES antigen separated by sodium dodecyl sulphate polyacrylamide gel electrophoresis (SDS-PAGE), which revealed reactions at 70 and 110 kd antigen. Determination of isotyping indicated that the monoclonal antibody was mouse IgG1. Antigen localization by fluorescent immunohistochemistry demonstrated that it reacted to the tegument, muscle, and parenchyma of the adult *O. viverrini*. The positive clones with high titers (clone KKU505) was subsequently expanded and used for inoculation of BALB/c mice to produce monoclonal antibody in ascetic fluid.

**References:**

1. Amornpunt S, Sarasombath S, Sirisinha S. Production and characterization of monoclonal antibodies against the excretory-secretory antigen of the liver fluke (*Opisthorchis viverrini*). Int J Parasitol. 1991;21(4):421-428.

2. Billings PB, Utsakhit N, Sirisinha S. Monoclonal antibodies against *Opisthorchis viverrini* antigens. Parasite Immunol. 1990;12(5):545-557.
