# Supplementary material for: Advances in the Diagnosis of Human Opisthorchiasis: Development of Opisthorchis viverrini Antigen Detection in Urine
Source: PLoS Negl Trop Dis. 2015 Oct 20;9(10):e0004157. doi: 10.1371/journal.pntd.0004157 (PMC4618926; doi:10.1371/journal.pntd.0004157)
Supplement: S1 Table — (DOCX) [file pntd.0004157.s002.docx]

**S1 Table. Diagnosis of *O. viverrini* by FECT and OV-ES assay of TCA-treatment samples in different sample sets.**

| **Phases of Study Development** | **Participants in Sample (N)** | ***O. viverrini* +ve** | |
| --- | --- | --- | --- |
|  |  | **FECT (%)** | **OV-ES assay (%)** |
| **1. Optimization** |  |  |  |
| Parasite negative | 10 | 0 (0) | 0 (0) |
| *O. viverrini* | 40 | 40 (100) | 40 (100) |
| **Total** | **50** | **40 (80)** | **40 (80)** |
| **2. Verification** |  |  |  |
| Parasite negative | 63 | 0 (0) | 28 (44.4) |
| *O. viverrini* | 125 | 125 (100) | 112 (88) |
| Other parasite* | 47 | 0 (0) | 12 (25.5) |
| **Total** | **235** | **125 (53.2)** | **152 (64.6)** |
| **3. Cross-reactivity** |  |  |  |
| *O. viverrini* | 97 | 97 (100) | 89 (91.7) |
| Other parasite** | 92 | 0 (0) | 3 (3.3) |
| **Total** | **189** | **97 (51.6)** | **92 (48.6)** |

*Other parasites included *S. stercoralis*, minutes intestinal fluke (MIF), hookworm, *Echinostoma* spp. and *Taenia* sp. The term “%” refers to the ratio of the number of individuals identified from the total number of individuals in each group.
