# Supplementary figures and images for: Advances in the Diagnosis of Human Opisthorchiasis: Development of Opisthorchis viverrini Antigen Detection in Urine
Source: PLoS Negl Trop Dis. 2015 Oct 20;9(10):e0004157. doi: 10.1371/journal.pntd.0004157 (PMC4618926; doi:10.1371/journal.pntd.0004157)

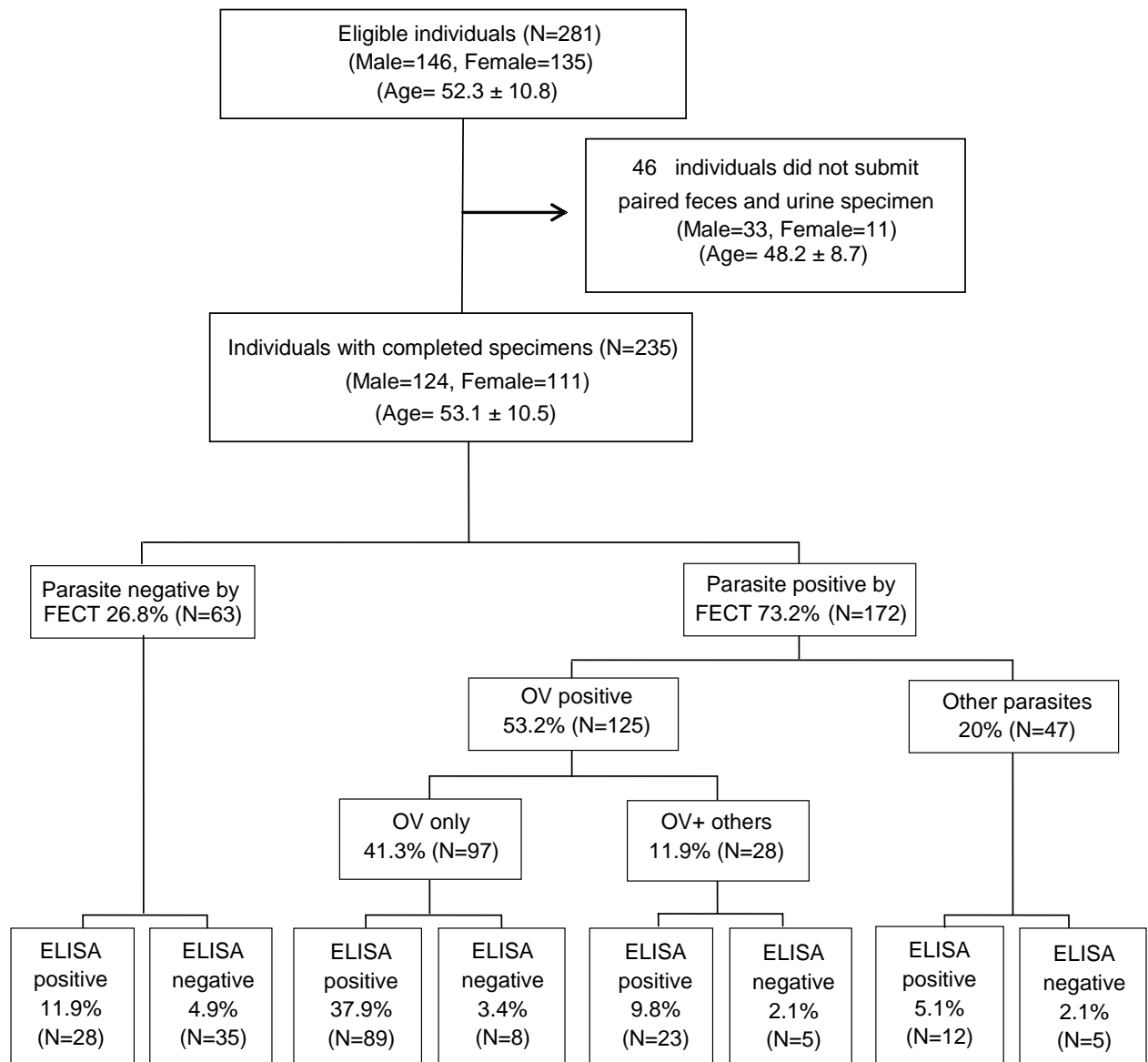

Supplement: S1 Fig — N refers to the number of participants. The abbreviation “OV” refers to O. viverrini. Other parasites include S. stercoralis, Minute intestinal fluke (MIF), Hookworm, Echinostoma spp. and Taenia sp. (PDF) [file pntd.0004157.s003.pdf]

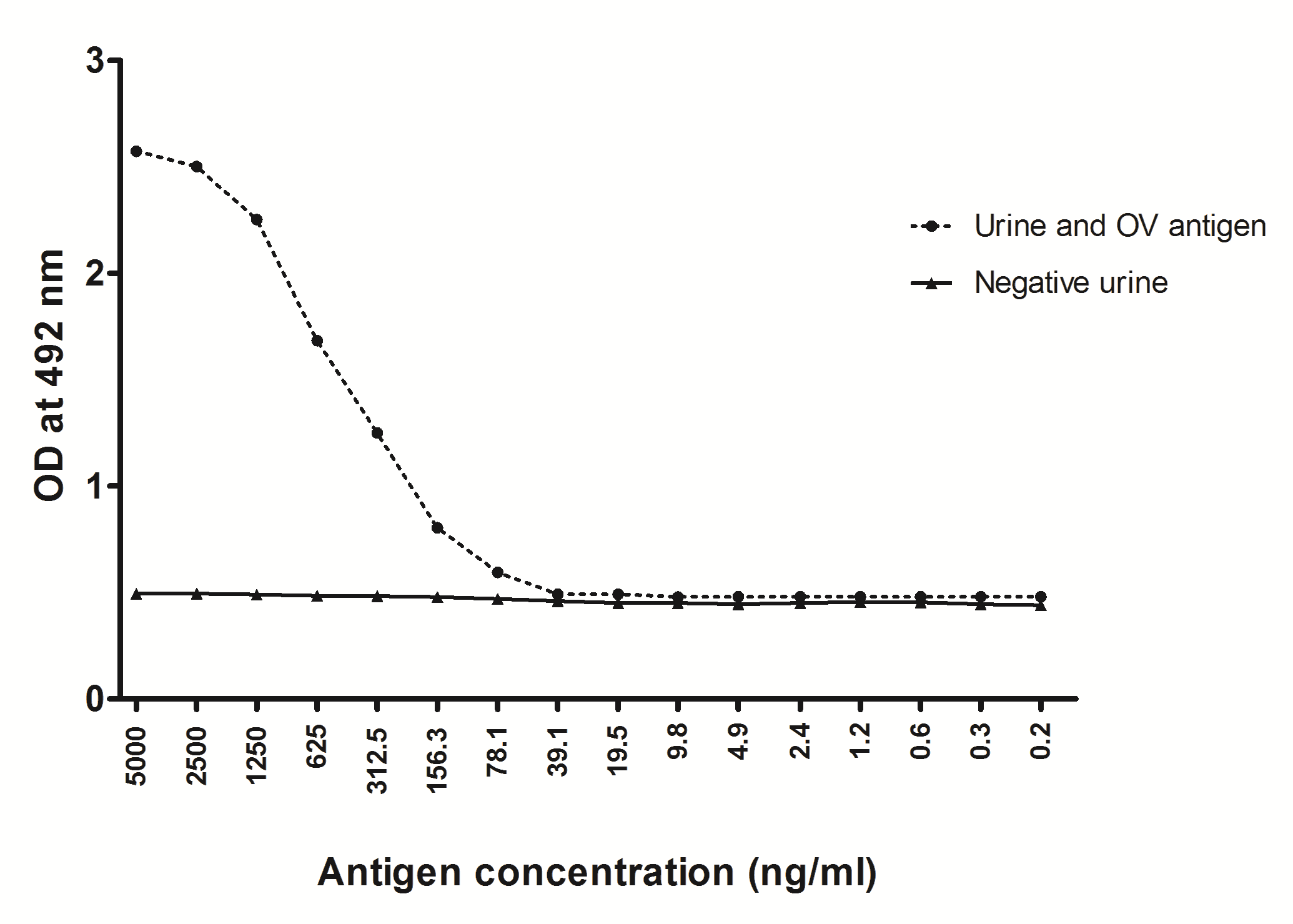

Supplement: S2 Fig — Data shown are optical density (OD) at 492 nm from OV-ES assay of TCA treated urine specimens against varying concentration of OV-ES antigen compared with negative control. (TIF) [file pntd.0004157.s004.tif]
